# “You cannot know if it’s a baby or not a baby”: uptake, provision and perceptions of antenatal care and routine antenatal ultrasound scanning in rural Kenya

**DOI:** 10.1186/s12884-015-0565-5

**Published:** 2015-05-29

**Authors:** Dorothy A Oluoch, Nancy Mwangome, Bryn Kemp, Anna C Seale, Angela Koech, Aris T Papageorghiou, James A Berkley, Stephen H Kennedy, Caroline OH Jones

**Affiliations:** KEMRI-Wellcome Trust Research Programme, Coast Centre for Geographic Medicine and Research, Kilifi, Kenya; Nuffield Department of Obstetrics & Gynaecology, University of Oxford, Oxford, UK; Centre for Tropical Medicine, Nuffield Department of Medicine, University of Oxford, Oxford, UK; Oxford Maternal and Perinatal Health Institute, Green Templeton College, University of Oxford, Oxford, UK

**Keywords:** Perceptions of antenatal care, Obstetric ultrasound scanning, Antenatal care timing, Gestational age, Confirmation of pregnancy, Sub-Saharan Africa

## Abstract

**Background:**

Antenatal care early in pregnancy enables service providers to identify and manage risks to mother and fetus. In the global north, ultrasound scans are routinely offered in pregnancy to provide an accurate estimate of gestational age and identify potential problems. In sub-Saharan Africa, such services are rarely available and women often delay initiating antenatal care. This study describes the uptake and provision of antenatal care in a rural Kenyan hospital and explores how pregnant women and healthcare providers perceived the provision of ultrasound scanning, following its introduction in an international foetal growth study.

**Methods:**

A descriptive study, using qualitative and quantitative methods, was conducted in Kilifi District Hospital, Kenya, between June 2011 and April 2012. In-depth interviews were conducted with 10 nurses working in the antenatal clinic (ANC) and 59 pregnant women attending ANC. Structured observations of 357 ANC consultations and 30 ultrasound scans were made.

**Results:**

Women sought antenatal care for information about the health of their baby and the protection provided by the ANC services. Uncertainty about pregnancy status contributed to delay in ANC attendance; more than 78 % of women were over 20 weeks’ gestation at their first visit. Healthcare workers found it difficult to detect pregnancies below 16 weeks gestation and, accurate assessment of gestational age below 20 weeks’ gestation could be problematic. Provision of services depended on the pregnancy being detected and gestational age assessed. The “seeing”, made possible through ultrasound scanning was perceived by pregnant women and healthcare workers to be beneficial: confirming the pregnancy, and providing reassurance about the fetus’ condition. Few participants raised concerns about ultrasound scanning.

**Conclusions:**

Uncertainty about pregnancy status and gestational age for women and healthcare providers is a key factor influencing timing of ANC attendance, contributing to delays and restricting early provision of ANC services. Ultrasound scanning was perceived to enhance antenatal care through confirmation of pregnancy status and enabling more accurate estimation of gestational age and the health status of the fetus. There is a need to make available more affordable means of pregnancy testing as a strategy towards encouraging early attendance, and delivery of antenatal care.

## Background

Each year, over a million women die in childbirth or from pregnancy-related complications. Almost all these deaths occur in low-income countries and more than half are in sub-Saharan Africa (sSA) [1–5]. In the early 1990s, against a background of evidence that birth outcomes can be improved if women seek antenatal care early in pregnancy, the World Health Organization (WHO) endorsed a package of Focused Antenatal Care (fANC) services [6]. Based around a series of cost-effective interventions delivered over four visits, the full benefits of fANC depend upon early presentation for: 1) the delivery of preventive interventions of proven efficacy; 2) the identification of women at increased risk of adverse pregnancy outcomes, who warrant close surveillance, and 3) establishing good relations between the women and their healthcare providers to encourage skilled delivery at birth [5–8].

According to the most recent estimates, over 71 % of women in sSA attend an antenatal clinic (ANC) at least once during pregnancy, although only 47 % achieve the recommended four visits, compared to 81 % in high-income countries [9]. Furthermore, most women in sSA first attend ANC during or after the second trimester [10–12] and, as a consequence, they do no benefit fully from the services offered and health care provided [13]. Several studies have found that late uptake of care is associated with higher maternal age and parity and lower economic status as well as misunderstandings surrounding the requirements of antenatal care [8–14].

A recent multi-country study (Ghana, Malawi and Kenya), of the factors affecting the uptake of fANC found a number of major barriers to the timely uptake of ANC services. These included: uncertainty about pregnancy status and gestational age (GA); logistical issues such as cost and distance, and supply side factors such as how service providers responded to women’s uncertainties and the messages given about the timing of ANC visits [14].

In high-income countries, women are able to address uncertainties about pregnancy status using home pregnancy testing kits, which are readily available and easily accessible. Similarly, ultrasound (US) scans are routinely used by healthcare professionals to estimate GA and assess foetal growth. Studies on women’s attitudes towards US in the UK and USA have found that they provide reassurance and help to promote healthy behaviours during pregnancy [15–18]. By contrast, in many countries in sSA, US is rarely if ever routinely used within the public sector and rapid pregnancy tests are comparatively expensive and often not available in public health facilities [19]. It has been argued that the versatility and relatively low cost of antenatal US in comparison to other imaging techniques could justify the case for its routine implementation in low-income settings [20].

In the Kenyan public sector, US scanning is seldom offered as part of routine fANC in district hospitals or the rural health facilities where the majority of pregnant women receive their antenatal care. Instead, GA is based on the date of a woman’s last menstrual period (LMP) or, if the LMP is uncertain/cannot be recalled, the symphyseal-fundal height (SFH) of the uterus, which is converted to weeks using standard dating charts [21].

In October 2011, an US service was established within the ANC clinic at Kilifi District Hospital (KDH) in rural coastal Kenya to assess GA and fetal growth for clinical and research purposes. The US service was part of the INTERBIO-21^st^ Study, a multi-country, multicentre study examining preterm birth and low birth weight syndromes across geographically diverse populations (www.interbio21st.org.uk).

The introduction of the service provided an opportunity to: 1) undertake a longitudinal study of antenatal care provision and use in a district hospital in rural Kenya and 2) investigate the perceptions and experiences of healthcare providers and pregnant women in a low-resource setting regarding the use of US in antenatal care and, more specifically, the effects of estimating GA accurately.

## Methods

### Study setting

The study was undertaken between June 2011 and April 2012 in the KDH ANC. The most recently available Demographic and Health Survey (DHS) data from Kenya, collected in 2008–9, indicate that 94.5 % of women in the coastal area make at least one visit to ANC during their pregnancy and that the median GA, by clinical estimates, at the first ANC visit in Kenyan rural areas is about 25 weeks [22]. A basic US has been available for pregnant women within KDH for ad-hoc clinical requests, but there has been no early pregnancy US service available to pregnant women since the 1990s [23].

### Data collection methods

**Fig. 1:**
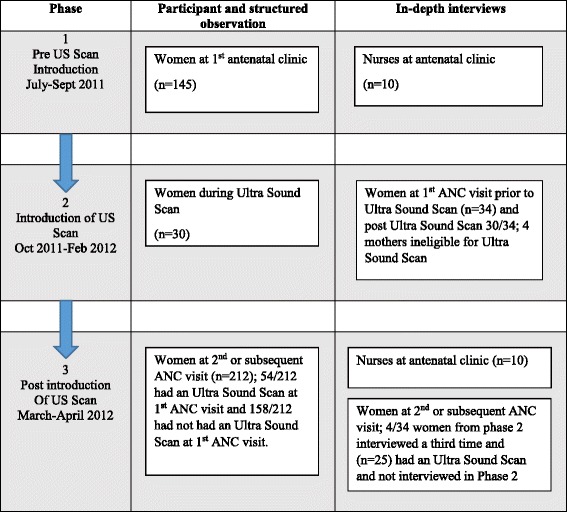
Data collection flow

Data were collected in three phases using a combination of participant observations, in-depth interviews and structured observations (Fig. 1).

Prior to the formal data collection, a trained research assistant spent six weeks in the ANC becoming familiar with the staff and gaining an understanding of the nature of service provision and the working structure of the ANC. During this familiarisation period, formal and daily informal interactions with the staff took place. The goal of the study was explained to the staff and this familiarisation process played a key role in building a good working relationship and rapport with the ANC team.

Phase 1: Data on care provision at the ANC were collected through participant observation, an ethnographic method in which researchers immerse themselves into the way of life of the population under study, participating and observing daily interactions [24]. The research assistants spent several hours each day for two months in the ANC observing and participating in non-clinical roles such as assisting with registration of clients, packing drugs and providing drinking water to the clients for taking their medication. Once the researchers were certain that the staff were comfortable with their presence, structured observations of service provision were undertaken each day for a further eight weeks. This period of participant observation took place in the ANC prior to the introduction of the US service.

Phase 2: Once the US service became available, we carried out structured observations using standard observation checklists to capture data on the US scan procedure as well as inviting women for interviews before and after their US scan.

Phase 3: Five months after the US service started, we carried out structured observations using a standard observation checklist to capture data on service provision at the second or subsequent ANC visit. In addition, we conducted follow-up interviews with women who had been interviewed and observed undergoing an US scan during their first ANC visit, as well as women who had been scanned but not previously interviewed. During this phase we also conducted follow-up interviews with the ANC nurses to elicit their views and experiences of the introduction of US scanning early in pregnancy.

Interviews were conducted by two trained research assistants in two languages (Kiswahili and Kigiriama (the local language) and were guided by standard topic guides.

Each interview took between 30–50 min and was conducted in a designated room within the hospital.

### Data analysis

Interviews were transcribed verbatim and translated into English by trained transcribers and verification of the transcripts was carried out by the research assistants. We developed a thematic framework based on the interview topic guides, which was then revised to include new themes emerging from the data. Following the construction of the coding framework, themes were indexed and segments of the transcripts sorted into the relevant categories using NVivo 9 software. Data from the observation checklist were entered into Excel for management and descriptive quantitative analysis.

### Ethical approval

This study was carried out in compliance with the Helsinki Declaration. Ethical approval was granted by the Ethical Review Committee of the Kenya Medical Research Institute (Protocol no. 1969). In addition, permission to conduct the study was obtained from the Medical Superintendent and Matron of the ANC. Written informed consent was obtained from the study participants after explaining the purpose of the study.

## Results

Fifty-nine women attending the KDH ANC and 10 staff who were involved in providing these services were interviewed. Of the 59 women interviewed, 34 (58 %) were interviewed at their first ANC visit before their US scan and 26 of these women were also interviewed immediately after their US scan. Four were then re-interviewed on one of their subsequent ANC visits (Fig. 1). Twenty-five women, who had been US scanned during their first ANC visit, were only interviewed at their second or subsequent ANC visit.

In Phase 1, we carried out observations of 145 first visits to the ANC. In Phase 2, structured observations of 30 US scans were conducted. In Phase 3, we carried out 212 observations of care provision at second or subsequent ANC visits. Of the 212 s or subsequent ANC visits observed, 54 (26 %) of the women had been scanned at their first visit, while the remainder had not.

### Gestational age at first ANC visit

Observations confirmed that the LMP was primarily used to estimate GA at KDH. Data were recorded by nursing staff or students, who would probe those women unable to recall LMP by referring to specific events or times of the month as an aid to recall.

After recording a menstrual history, each woman underwent an individual consultation with a member of the nursing team. By abdominal palpation, the healthcare provider estimated the lie and presentation of the fetus and the height of the uterine fundus. If this was below the umbilicus, then the number of fingers on the left hand between the fundus and the umbilicus would be counted and used to estimate GA by subtracting 2 from 20 for each finger-breadth below the umbilicus; the reverse was applied when the fundus was above the umbilicus. From our observations of second and subsequent ANC visits, more emphasis was placed on the SFH estimation of GA rather than the LMP even if this was certain; the two were rarely compared.

On several occasions, when reviewing previous GA estimates to assess progress of a pregnancy, nursing staff would query the previously reported findings, especially if the current finding was lower than had been recorded for the previous months.

From the observation data, and from informal conversations held throughout the study, it became apparent that the nursing staff found it difficult to estimate GA accurately by SFH for pregnancies less than 20 weeks’ gestation, particularly when no tape measures were available. Of the 34 women interviewed at the first ANC visit, the nurses were unable to estimate GA by SFH in the five out of 12 pregnancies that were less than 16 weeks’ gestation. For these five women, an outcome of ‘no mass’ was recorded in the GA column of the mother-child booklet. Importantly, the data from observations at the first ANC visit suggest that in cases where nursing staff were unsure of the pregnancy status by their own assessment [13/145], women would not be provided with the recommended intervention for that visit, and were asked to return at a later date instead. Pregnancy test kits are not routinely available at the antenatal clinic at KDH, thus those whom pregnancy status was uncertain were not offered the pregnancy testing service.

Participants in the INTERBIO 21^st^ Study were informed by the utra-sonographer of their GA as assessed by US. Not surprisingly, where there was significant variation among the dates provided by the different methods, women expressed concerns. In two such cases where the difference was more than 2 weeks, the women expressed feelings of uncertainty, stating that they would wait to see when they would deliver as a means to determine which method was “telling the truth”.

### Timing of first ANC visit; Women’s reasons for booking

Using the data from the health workers’ assessments of GA based on SFH, more than three quarters (78.6 %) of the 145 women attending their first ANC visit were over 20 weeks’ gestation (Table 1). The GA for the first visit of the 34 women enrolled in the INTERBIO-21^st^ Study was lower due to the nature of the study selection criteria. However, 24 % of the women interviewed were over 20 weeks’ gestation at their first ANC visit and 56 % were over 16 weeks’ gestation as assessed by SFH (Table 1).

**Table 1:** Gestational age among women at first ANC visit, assessed by fundal height measured by hand

Gestational age (weeks) measured by hand	Number of women observed at first booking n = 145 (%)	Number of women interviewed at first booking n = 31* (%)
<16	13 (9)	12 (35)
16-20	18 (12)	11 (32)
21-28	71 (49)	8 (24)
29-32	25 (17)	0 (0)
>32	18 (12)	0 (0)

*(records missing for three women)

All women interviewed (whether at their first or second ANC visit) were asked to discuss the reasons for initiating care. The most common reason for attendance (18 /59) was that, problems, such as abdominal pain or general feelings of being ‘unwell’, prompted the visit.

“I started at three months because I had problems”. C012

“I had problems, the skin changed and then I was not eating food eeh and then the body was completely thin eeh”. C019

“The right time” was referred to as the second most common reason for visiting (16/59). This group of women thought there was sufficient time remaining in the pregnancy for them to receive all the necessary interventions.

“It’s because I did not have any complications/problems and what I know you are supposed to visit the clinic at least four times so I estimated from five months there I can start”. A001

“When you start early you will go for many times. When you are one month old and start the clinic immediately you normally come for many times”. A002

The objective of the fANC model for low-risk women is to provide all the necessary interventions spread over four visits; however, it was clear from our observations that once booked, women were routinely told by the health workers to return for appointments on a monthly basis, regardless of their GA at the first visit. Most women were aware of the recommended four ANC visits and, based upon their experiences at KDH, assumed that these should be at monthly intervals from the first visit onwards, hence the ‘right’ time to initiate ANC was at 5 months to allow for a total 4 visits before giving birth.

The third most frequently mentioned reason (10/59) for initiating ANC was that this was the earliest point at which they, themselves, felt certain they were pregnant; they had waited until fetal movements were felt, or until it was obvious they were pregnant:

“I did not start early because you cannot know if it’s a baby or it’s not a baby because you might say it’s a baby then later on after a month you get your monthly period as usual so you have to check first whether it’s a baby or it’s just getting a break and get my periods later on”. W008A

“Five months, there is no apparent reason for this, I just wanted my pregnancy to grow a bit and for the baby to develop a bit”. W010A

Finally, logistical issues were cited as influencing the timing for 8/59 women. They reported that they had been preoccupied at home or had been travelling and so had been unable to attend. Four of these women reported waiting until sufficient funds were available to pay for the services:

“I was looking for money”. W003A

“I didn’t have money because I wasn’t able to walk and I didn’t have that fare to come here”. W007A

The remaining three women were influenced by the advice from previous healthcare providers (n = 2) or from their husband (n = 1).

“I just decided to start early even the doctor said you can start with two months”. W009A

### Providers’ views on ANC bookings at KDH

Nursing staff were asked their opinions about the timing of ANC initiation. Overall, the consensus was that a woman should attend as soon as she is aware of her pregnancy. For 9 out of the 10 nurses this was at, or before, 16 weeks’ gestation as this would provide adequate time to detect problems with the pregnancy and ensure that all the required interventions could be delivered. This is the time recommended by the Kenyan ANC guidelines. Whilst the nurses expressed these views during the interviews, it was clear from the ANC observations, that they found it difficult to assess GA by SFH in pregnancies below 20 weeks’ gestation. We repeatedly observed instances where the measured SFH was questioned, a second opinion requested from a colleague, and, where a second opinion was unavailable, the SFH was recorded as “no mass” with GA subsequently assigned by LMP or not at all.

When there was uncertainty about GA, which was often the case, the administration of suphadoxine-pyrimethamine for malaria prophylaxis and tetanus toxoid were withheld due to safety concerns. Only once the SFH could be assessed with certainty to be at least 20 weeks’ gestation were these interventions provided. Interestingly, despite the recommendations for the timing of the first visit in the guidelines, a minority of the nurses interviewed (n = 2) were explicit about these problems and considered 20 weeks’ gestation as appropriate since this was the time when the uterus is easily palpable and the time at which the first dose of suphadoxine-pyrimethamine is indicated.

Nursing staff suggested that early presentation for care was more common amongst younger women, with some level of education and for those women expecting to conceive. Older, multiparous women were said to be more likely to book later, which was considered by nursing staff to reflect a desire to avoid repeated ANC visits, because of cultural or financial constraints. It was also felt that women with previously successful pregnancies presented late.

### Women’s reasons for attending ANC & perceptions of routine US in ANC

Two broad themes for attending ANC emerged from our data; reassurance and protection (Table 2).

**Table 2:** Mother’s perceptions of the benefits of ANC

Benefits of ANC		Number of women n = 34 (%)	Number of women n = 25 (%)
Reassurance	Checking health of baby.	33 (97)	20 (80)
	Confirming pregnancy.	3 (9)	1 (4)
Protection	Safe-guarding health of mother and baby: testing and treating for example tetanus immunisation, malaria prevention.	13 (38)	2 (7)
	Counselling	7 (21)	0 (0)

### Reassurance

Almost all of the 59 women interviewed reported that their primary reason for attending the ANC was to be reassured about the health of their fetus. A few also mentioned that they were keen to confirm whether or not they were pregnant (Table 2). They described being reassured after using ANC services and suggested that only by attending the ANC was a woman able to be informed of the wellbeing of her fetus.

“I come for the clinic, I come to be able to know how am also progressing and the baby as well”. W021A

Among those women who were interviewed prior to receiving an US scan the majority (21/34) reported no concerns about the process. Amongst the 13/34 who reported concerns, 10 said they were worried that, rather than contributing to protection, the US might harm the mother or fetus (Table 3). However, undergoing the US scan, seeing how it was conducted and being provided with adequate information enabled them to deal with these concerns.

**Table 3:** Benefits and concerns of US Scan; women interviewed before US scan

Mothers’ views of benefits and concerns before US scan		Number of women n = 34 (%)
Benefits of USS		
Reassurance	Knowledge of health of baby	30 (88)
	Seeing	22 (64)
	Confirmation of pregnancy	4 (12)
	Confirmation of gestational age	13 (38)
Information	Number of babies	6 (18)
	Sex determination	1 (3)
Concerns of USS		
Harming the baby	The machine uses rays which could affect the baby	5 (15)
Harming the mother	The machine used during the procedure could hurt the mother	5 (15)
Uncertainty of outcome	Not being sure of state and condition of the baby and not knowing real reason for being asked to undergo US scan	3 (9)

A further three women were concerned that the primary reason for being offered a scan was an assumed problem with their pregnancy.

“I thought that an expectant mother is not supposed to have an US, unless there is an emergency or sickness or maybe there is a certain problem”. W033A

“At first my heart was beating very fast, because I did not know what the outcome would be, I was anxious in the beginning”. W10B

This perception was consistent with how scans have historically been offered by the clinical service at KDH. That is, US is only offered when a problem is suspected. Amongst the 25 women who had an US scan but who were interviewed during a second or subsequent ANC visit, 22 reported that they had not had any concerns, mainly as a result of the dialogue between clinical staff and themselves during the procedure. Of those, three reported that they had been concerned: two said they had been concerned that the procedure might harm the baby and one that it might harm them (Table 4).

**Table 4:** Benefits and concerns of US Scan; women interviewed after scan or at subsequent ANC visit

Mothers’ views of benefits and concerns after US scan		Number of women (%) N = 30	Number of women (%) N = 25
Benefits of US scan			
Reassurance	Knowledge of health of baby	30 (100)	22 (88)
	Seeing	30 (100)	18 (72)
	Confirmation of pregnancy	4 (13)	17 (68)
	Confirmation of gestational age	22 (73)	7 (28)
Information	Number of babies	1 (3)	2 (8)
	Sex determination	2 (7)	1 (4)
Concerns of US scan			
Harming the baby	The machine uses rays which could affect the baby	0 (0)	2 (8)
Harming the mother	The machine used during the procedure could hurt the mother	0 (0)	1 (4)
Uncertainty of outcome	Not being sure of state and condition of the baby: implications of low lying placenta	3 (10)	1 (4)

“Others they didn’t like the scan, because they say they use harmful rays and it affects the baby”. C016

Among the 55 women scanned who were interviewed (30 immediately after the scan and 25 at their second or subsequent ANC visit), all perceived that US provided significant additional benefits in term of reassurance. They said that the scan gave healthcare providers more details about the baby’s health and enabled a better assessment of the state, progress and condition of the baby within the uterus (Table 4). In addition to enhancing the providers’ knowledge thereby enabling them to provide better care, the majority of the women also said that US gave additional reassurance because they were able to ‘see’ their baby (table 4).

“I also informed her (sister in law who is also pregnant) about the scan and she said it is good because you will know how the baby is in the womb you will see by yourself, that day I saw the baby and I informed her and I told her I have seen the baby she said she would also like to the see the baby the way it is”. 1C: 011

“I was afraid [before the US], you know it is like aahh.. you are just walking but you don’t know what’s inside,[getting on with life without knowing the progress of the pregnancy], so I was afraid but later on I gathered some courage and I decided that it is better for me to see, in case it [the pregnancy] is okay then that’s fine, if it is not, then I just know what I have carried in the womb, but I was very much happy to see what am carrying, [the baby in the womb] is fine and has no problems”. WB006

“I was pleased because I saw the baby is safe and so I won’t be worried any more”. WB021

Several participants also said that US was beneficial as multiple pregnancies (9/59) could be detected. Interestingly, some (4/59) mentioned fetal sex detection as a potential benefit even though it was made absolutely clear to all women having an US scan that this information was never revealed.

Although almost all the women interviewed immediately after the scan at the first ANC visit (27/30) expressed no concern at that point. For the three women who did express some concern, this was as a result of learning about the presence of placenta praevia.

“Yes am worried because I have been informed about the wrong position of the placenta, [placenta praevia], because of this news i must be worried now eeh”. 012B

“I wasn’t happy when I was told that the placenta wasn’t good, it’s positioned wrongly, and as far as what the doctor has advised me, it is bad if it continues like that, the placenta will come out first then the baby to follow which is dangerous. I didn’t know what kind of danger it was either I die or the baby dies so am not well enlightened”. 019B

### Protection

Treatments provided by ANC services were considered by mothers to be protective for both themselves and the fetus. It was recognised that prophylaxis against malaria and tetanus, as well as prevention of mother to child transmission of HIV, were important benefits of ANC care. The need to “test” for the presence of known causes of harm was also considered to be important for the mother and fetus, particularly if a treatment was available:

“I take it to be important for pregnant mothers because you are tested; everything is tested so they protect you well from diseases and also so that you know before you get the baby so as to protect the health of the baby”. (tested for HIV) W027A

Protection was perceived in terms of access to advice from the nursing staff about issues during pregnancy and the postnatal period:

“A pregnant woman is supposed to come to the clinic where she can get the advice from the nurses and if she can follow the advice given, she can be able to maintain her pregnancy well until she delivers well”. W016

The US scan was seen to add to this protection as it allowed the healthcare providers to identify any problems more easily and guide them regarding the course of action to take or advice to give.

“I don’t know the state of the baby now, so that picture can show how the baby is. If there is any problem it will be known early and they [healthcare providers] can advise more on what to do for the benefit of the baby”. A005

### Provider perceptions of US and ANC services

Amongst the nursing staff, US scanning was considered to support provision of care. It was generally accepted that US could provide a clear depiction of the uterus and its contents, and that the ‘clearer report’ provided by the scan was more reliable than standard clinical assessment.

“Clinical advantage is that the results might show something that needs follow up and this facilitates booking for outpatient clinic, if the US scan was not done this would not be the case, we maybe will just be progressing blindly”. N008

Some nurses also eluded to work satisfaction especially when women, who after palpation were thought to have a problem such as unclear heartbeat or wrong presentation/lie, could access an US scan soon after the palpation, on the same clinic visit. This they felt helped them rule out any underlying abnormalities or problems early enough and was, therefore, important in contributing to the prevention of maternal and neonatal mortality.

Nurses felt that the study made it possible for women to access US scans at no cost, which was not the case in the past. They mentioned that a number of women who previously needed an US scan could not afford to pay the fees.

“Again we are at peace, because you find that you are comfortable once the USS has been done and foetal heart rate confirmed and you confirm that the fetus is viable and there is no problem, so at least you let the mother go home knowing everything is fine as at the time she visited the clinic, so in case something happens you can trace when the problem started. So this makes follow up easier”. N009

Nurses also mentioned that US was helpful to them in determining accurate GA and expected delivery dates especially when the LMP was unknown.

“The results are useful because you know there are some mothers who do not know their LMPs, again the results are useful to get the diagnosis and also to know when she will have the baby”. N002

However, the majority of the nurses (nine out of the 10 interviewed) voiced concerns about the feasibility of maintaining routine US scanning as part of regular ANC practice, without the support of the research staff. In their view, routine scanning would be realized only if trained staff are made available and if strategies are put in place to ensure the maintenance and regular servicing of the US machines. The research team had provided training for the ultra-sonographers both in US scanning and counseling, and was also responsible for the purchase and the maintenance of the machine. From the interviews with the nurses, there was a general feeling that the Maternal & Child Health (MCH) department was understaffed; a factor that they felt could impede effective counseling and information giving to mothers during consultations.

In addition, most of the nurses felt that routine US scanning at the ANC would be more feasible and easily implemented if the service could be integrated within the ANC rather than in the Radiography Department, which serves all patients in the hospital. In their view, having a designated ANC US machine within the MCH department would reduce the waiting time for the pregnant women and help ensure that the service was available whenever the ANC was open.

## Discussion

In this study we investigated the uptake, provision and perceptions of antenatal care among women. We also explored the effect of the introduction of routine US scanning on the perceptions of the women and healthcare providers in a rural district hospital on the Kenyan coast. At KDH, the majority of women present for ANC during late second trimester of pregnancy, or beyond, which is consistent with data elsewhere in sSA [25–28]. Several studies have suggested that late attendance can primarily be attributed to socio-economic, demographic and logistical factors as well as individual beliefs and perceptions regarding the appropriate time to initiate ANC visits [26–30]. Our results, plus those of two other recent studies, [14, 15] suggest that uncertainties about pregnancy status and GA among both pregnant women and healthcare providers are key factors influencing the late initiation of antenatal care.

The first indication of a pregnancy for most women is missed menstruation, but in an era of long acting contraceptives, it is sometimes difficult to distinguish whether the absence of menstrual bleeding is the on-going effect of contraception, or a sign of pregnancy. In addition, other factors such as nutritional status can interfere with the menstrual cycle and this area of Kenya is very prone to drought and food shortages [31], which not only affect nutritional intake but also cause significant stress and place additional burdens on women [32]. In this study, women reported that they first became aware they might be pregnant when they appeared to miss a menstrual period, but due to the uncertainty, many preferred to wait until they felt the movement of the fetus before they started to attend the ANC. Furthermore, without diagnostic technologies routinely available to confirm a pregnancy, the providers of care themselves often found it difficult to confirm pregnancy in women below 20 weeks’ gestation. The inaccuracy of using the date of the LMP and SFH measurement as methods for determining GA is well documented [33] but its impact on the behaviours of ANC providers in resource-poor settings in sSA has only recently been raised as a potentially important issue affecting the uptake and provision of antenatal care [28] [34]. In the current study, when the healthcare providers were unsure of pregnancy status they were unwilling to provide some of the key preventive services that rely on the accurate assessment of GA. For instance, intermittent prevention of malaria in pregnancy (IPTp) should be provided four times in a pregnancy, with the first dose to be administered after 16 weeks’ gestation. As has been found in other studies [28, 34], where nurses were unsure if a woman was pregnant or if they had reached 16 weeks’ gestation, the woman was asked to return to the clinic the following month to have the pregnancy confirmed and to receive the necessary interventions. Data from this and other studies suggest that pregnant women share their experiences of ANC [14] and we hypothesise that women’s perceptions of the most appropriate time to initiate antenatal care is likely to be influenced by their experiences of nurses telling some women who make their first ANC visit at under 20 weeks’ gestation to go home and return at a later date as they are unable to confirm their pregnancy.

In high-income countries, US is routinely used as a tool to date pregnancies accurately and check for abnormalities and multiple pregnancies. A systematic review of the literature on women’s views of antenatal US found that there were few published data from low-income settings [19]. However, the data that exist suggest that in these settings, as elsewhere, where the staff and women were well informed about the process and what it could or could not achieve they were very happy to receive an US scan and enthusiastic about the potential benefits [19]. By contrast, where there was little or no explanation of the process, US was associated with significant psychological stress and anxiety in pregnant women [19]. A recent study of women’s views conducted in western Thailand where routine US scanning has been offered to migrant workers and refugees from Burma since 2001 had similar findings [20]. The authors reported that the majority of providers and pregnant women perceived that antenatal US improved patient safety, but some women who had not been given sufficient information, or had not properly understood the information provided, expressed anxiety about the process [20].

In our study, the US service was introduced as part of a research project and, as such, each pregnant woman was provided with detailed information about the process and potential outcomes, and given the opportunity to ask questions and refuse to participate if they had concerns. The risks and benefits of the US scans were explained and they were informed that they would not be told the sex of the fetus. They were also told about the possibility of the US scan revealing underlying abnormalities, and that if an abnormality was detected, referral for treatment would be made to KDH. We found that women appreciated the information given to them before undergoing the US scan which they reported enabled them to deal effectively with the stress and anxiety they might have felt undergoing an unfamiliar procedure. In addition, since most women had not had an US scan before, the ultrasonographers explained step-by-step to the women during the scan what they were doing and why. This interactive environment enabled the women to ask questions during the US scan. Verbal and non-verbal communication during the US scan was vital in building reassurance; however, as was found in the Thai study [20] women’s trust in the service providers at the ANC helped to enhance perceptions of the safety of the procedure. The women’s subsequent general satisfaction with the process was enhanced by their perception of the manner in which they were treated by the ultrasonographers. They particularly valued the interactive nature of the consultation and being allowed to see the fetus on the screen. At the end of each US scan, a photo of the fetus was attached to the mother-child booklet. Overall, there were very few cases of concerns after the scans and this could be attributed to women having been adequately prepared and informed about the procedure prior to scanning and not many abnormalities being identified. The service providers interviewed in our study also perceived counselling and information giving to be vital for routine antenatal US acceptability and feasibility.

When the appropriate counselling and information are provided, US scanning was viewed by the majority of pregnant women and the providers of care as significantly enhancing the value of antenatal care. The principal reasons that women gave for attending ANC was to receive information (reassurance) about the health status of their fetus, and to identify and help prevent any potential problems. The ability of US to provide a picture of the fetus enabled the women themselves to ‘see’ the baby and allowed the providers to diagnose any potential problems more accurately, reinforcing the reassurance and augmenting the potential protection of routine antenatal care. For example, in common with the results from Thailand and elsewhere [19, 20], our study found that women perceived US to be beneficial in the early detection of potentially life-threatening complications in pregnancy such as wrong position of the baby and placenta praevia. Women who had either been informed of a suspected problem after palpation in the ANC or those who had been informed of a low lying placenta after US scanning expressed anxiety and concern. However, they were grateful that both they and the healthcare providers were now aware of the problems and able to take steps to help mitigate risks. Early detection of such problems was perceived by both the women and the healthcare providers to be key in the management and care of pregnancy and childbirth and, as was found in Thailand, they appreciated the greater certainty in diagnosis that US provides. Overall the responses of the pregnant women and service providers who took part in this study suggest that US contributed to the quality of care at the ANC and could potentially increase early ANC attendance by facilitating the confirmation of pregnancy and accurate estimation of GA for pregnancies less than 20 weeks’ gestation, and through enabling a more accurate ‘picture’ of the health status of the fetus. Routine US scanning in pregnancy helps address key concerns of both pregnant women and healthcare providers but its potential in low-income settings is dependent on the effective implementation of services. In this project, the costs in terms of equipment, maintenance, space and staff to operate the machine were covered by the INTERBIO-21^st^ Study and it is unlikely that in a hospital where shortages of basic equipment such as gloves and thermometers are recurring problems that the funds would be available to provide a routine US scanning service within the County budget. However, we found that uncertainty about pregnancy status and GA both for the women and service providers is a key factor influencing the timing of ANC attendance. As a strategy towards encouraging early attendance more affordable modes of ascertaining pregnancy status, such as the provision of free pregnancy testing kits at ANC to test and confirm pregnancy for women who present early for care could be made available. While such kits can help confirm pregnancy there is still a need to develop low cost, low maintenance imaging technology for use in settings where funding and staffing pose huge challenges. The results of this and other studies suggest that the demand is there and that such technology has the potential not only to improve the quality of antenatal care but also facilitate its early uptake

This longitudinal study of antenatal care provision and uptake over 11 months in a rural Kenyan district hospital describes the complex interplay of factors that underlie the utilisation of antenatal care in this setting and highlights the importance of the relationship between the perceptions and practices of providers and the care seeking practices of pregnant women. A limitation to this study is that it was conducted within a research setting; therefore, some of the responses may have been influenced by the research itself as well as local perceptions of research activities in the area.

## Conclusion

We found that uncertainty about pregnancy status and GA both for the women and service providers is a key factor influencing timing of ANC attendance and appeared to contribute to delayed ANC initiation. US scanning introduced with attention paid to information sharing was perceived by both the women and providers to enhance antenatal care through confirmation of pregnancy status and enabling more accurate estimation of GA and the health status of the fetus. There is a need to make available more affordable modes of ascertaining pregnancy status as a strategy towards encouraging early attendance. It is also vital to put in place measures that will ensure sustainability of these services particularly in low-income settings where funding and staffing pose a huge challenge to the existing health systems. A further major contribution of this study is the knowledge that it adds regarding the perceptions of pregnant women and service providers on routine antenatal US in low-income settings.

## Abbreviations

ANC: Antenatal clinic
sSA: Sub-Saharan Africa
WHO: World Health Organization
fANC: Focused antenatal care
GA: Gestational age
US: Ultrasound
LMP: Last menstrual period
SFH: Symphyseal-fundal height
KDH: Kilifi district hospital
DHS: Demographic and health survey
HIV: Human immunodeficiency virus
MCH: Maternal & child health
KEMRI: Kenya Medical Research Institute
